# Implication of the Vaginal Microbiome in Female Infertility and Assisted Conception Outcomes

**DOI:** 10.1093/gpbjnl/qzaf042

**Published:** 2025-05-05

**Authors:** Xiuju Chen, Yanyu Sui, Jiayi Gu, Liang Wang, Ningxia Sun

**Affiliations:** Department of Reproductive Medicine, Second Affiliated Hospital of Naval Medical University, Shanghai 200003, China; Department of Reproductive Medicine, Second Affiliated Hospital of Naval Medical University, Shanghai 200003, China; Department of Reproductive Medicine, Second Affiliated Hospital of Naval Medical University, Shanghai 200003, China; Department of Reproductive Medicine, Second Affiliated Hospital of Naval Medical University, Shanghai 200003, China; Department of Reproductive Medicine, Second Affiliated Hospital of Naval Medical University, Shanghai 200003, China

**Keywords:** Vaginal microbiome, Infertility, Pregnancy outcome, Assisted reproductive technology, *Lactobacillus*

## Abstract

The rise in infertility rates has prompted research into the impact of vaginal microbiota on female fertility and the success of assisted reproductive technology (ART). Our study aimed to compare the vaginal microbiome between fertile and infertile women and explore its influence on ART outcomes. Vaginal secretion samples were collected from 194 infertile women and 100 healthy controls at Shanghai Changzheng Hospital. The V3–V4 region of the 16S rRNA gene was amplified using polymerase chain reaction (PCR). A machine learning model was applied to predict infertility based on genus-level abundance, and the PICRUSt algorithm was employed to predict metabolic pathways related to infertility and ART outcomes. The results showed that infertile women exhibited a significantly different vaginal microbial composition compared to healthy controls, along with increased microbial diversity. Notably, the abundance of *Burkholderia*, *Pseudomonas*, and *Prevotella* was significantly elevated in the vaginal microbiota of the infertility group, while that of *Bifidobacterium* and *Lactobacillus* was reduced. Among infertile women, those with recurrent implantation failure (RIF) showed even higher vaginal microbial diversity, with specific genera such as *Mobiluncus*, *Peptoniphilus*, *Prevotella*, and *Varibaculum* being more abundant. Eleven metabolic pathways were identified to be associated with both RIF and infertility, with *Prevotella* showing stronger correlations with these pathways. This study elucidates differences in vaginal microbiome between healthy and infertile women, providing novel insights into how vaginal microbiota may impact infertility and ART outcomes. Our findings underscore the importance of specific microbial taxa in women with RIF, suggesting potential avenues for targeted interventions to improve embryo transplantation success rates.

## Graphical abstract

**Figure:**
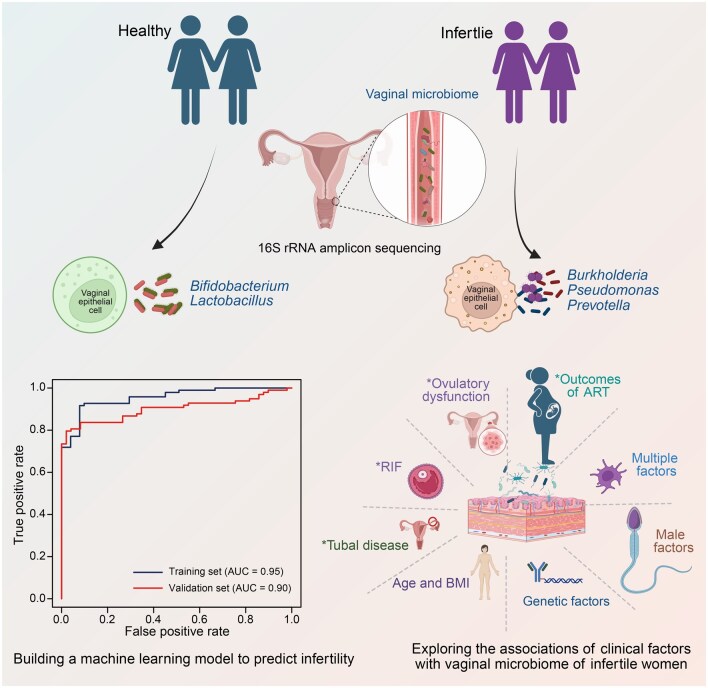

## Introduction

Infertility, defined as the inability to achieve a clinical pregnancy after 12 months or more of unprotected sexual intercourse, is a common challenge affecting approximately 15% of couples worldwide [1,2]. The proportion of couples affected by infertility is on the rise. The etiology of infertility is often complex and diverse and includes female, male, and unexplained factors. Female infertility alone accounts for 40% of these cases, with ovulation disorders, uterine or cervical issues, tubal alterations, endometriosis, immune factors, and pelvic infections being the primary causes [3]. Recent advancements in vaginal microecology and microbial detection technology have revealed a potential link between vaginal microecological imbalance and infertility, providing new insights into this complex condition.

The Human Microbiome Project has revealed that approximately 9% of the human microbiome originates from microorganisms inhabiting in the female genital tract (FGT) [4]. This microbiome plays a pivotal role in maintaining homeostasis, defending against pathogens, and potentially influencing fertility [5]. The vaginal microbiota, in particular, is typically dominated by *Lactobacillus* species [6], promoting a low-diversity environment. Conversely, a more diverse vaginal microbiota, characterized by the presence of various strict and facultative anaerobes, is described as vaginal dysbiosis [7]. Vaginal dysbiosis may increase the risk of infections, diseases, reproductive issues, and adverse pregnancy outcomes [8–10]. Assisted reproductive technology (ART) is a set of procedures designed to overcome infertility and achieve successful pregnancy [11]. Despite its widespread use for almost 40 years [12], the clinical pregnancy rate following ART remains stagnant, ranging from 30% to 40% [13].

Furthermore, recurrent implantation failure (RIF), as defined by Coughlan and colleagues [14], is a significant factor limiting ART success rates. Despite a growing body of literature on RIF, a universally accepted definition and standardized diagnostic and treatment protocols for RIF have yet to be established. The cause of RIF remains a mystery, and few studies have explored the association between reproductive tract microbiota and embryo implantation. Given the important role of the vaginal microbiome in maintaining homeostasis and affecting fertility, there has been increasing interest in exploring its relationship with ART outcomes. Multiple studies have provided evidence indicating that vaginal dysbiosis, characterized by alterations in the vaginal microbiota, is significantly associated with reduced success rates in *in vitro* fertilization (IVF), increased susceptibility to aneuploid pregnancy loss, and obstetric complications such as preterm prelabor rupture of membranes (PPROM) and preterm delivery [15–17]. The mechanisms by which the vaginal microbiome influences infertility and ART outcomes remain poorly understood. Although research has indicated that alterations in the vaginal microbiome can influence pregnancy, its precise role in infertility and ART success remains unclear.

In this study, we aimed to elucidate differences in vaginal microbiota distribution between fertile women of childbearing age and those experiencing infertility. To this end, we investigated the microbial composition within the infertile population and its association with ART outcomes. Additionally, we categorized a subset of infertile patients into RIF and non-RIF groups. This classification allowed us to explore microbial composition differences between these two groups and to assess whether specific microbial genera may serve as potential therapeutic targets for addressing ART failure.

By delving into this research, we hope to gain deeper insights into the potential role of the vaginal microbiota in fertility-related issues. Ultimately, these insights could contribute to more effective infertility treatments and improved reproductive outcomes, paving the way for more targeted therapeutic interventions in the future.

## Results

### Baseline characteristics of female patients with infertility and healthy controls

**Figure 1:**
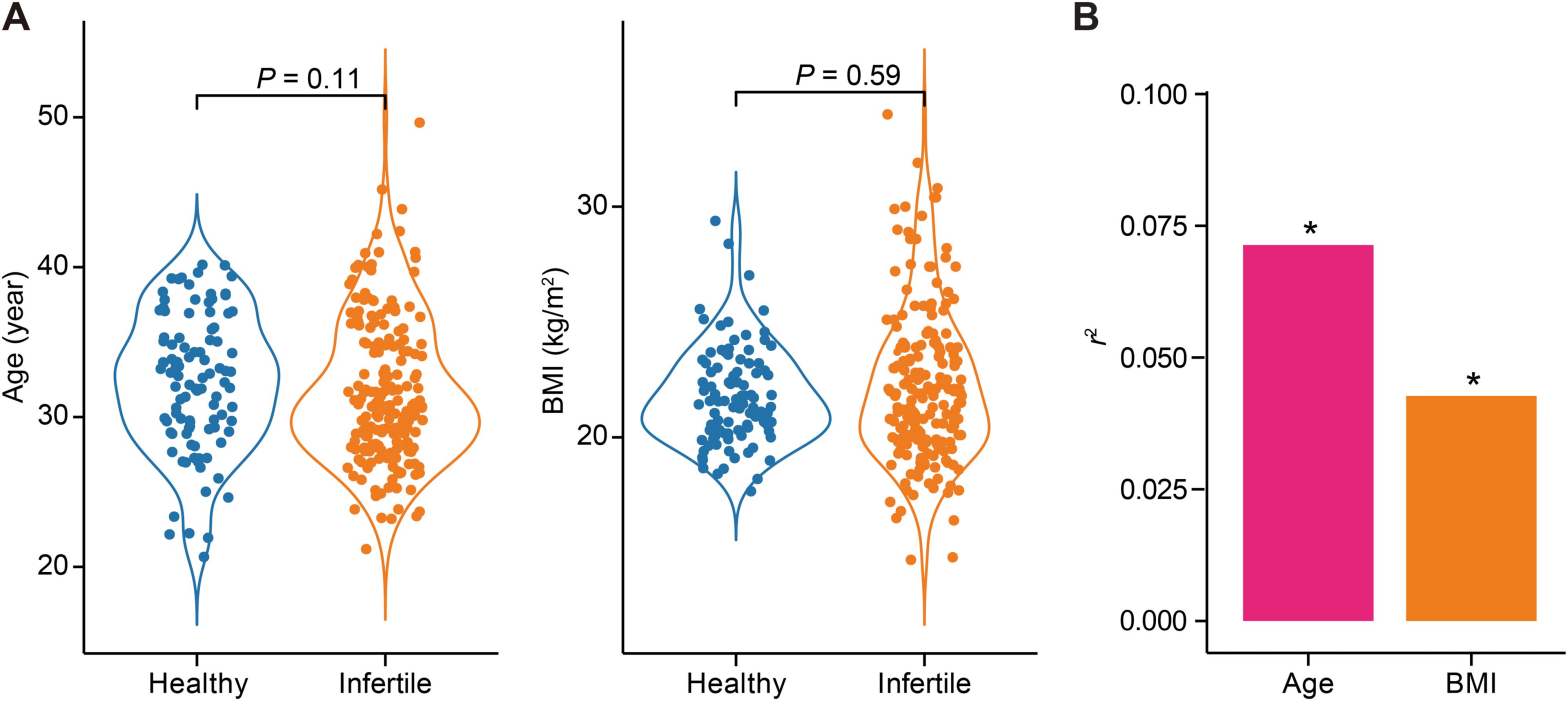
Comparison of age and BMI between healthy and infertile groups and their contribution to bacterial abundance **A**. Violin plots showing the distribution of age (left) and BMI (right) in the healthy and infertile groups. Statistical significance was assessed by Wilcoxon rank-sum test, with corresponding *P* values indicated above each plot. **B**. Bar plot displaying *r*^2^ (the strength of the correlation between the two sets of variable combinations) for the contribution of age and BMI to bacterial abundance, highlighting their relative explanatory power. *, *P* < 0.05 (Wilcoxon rank-sum test). BMI, body mass index.

We first compared the baseline characteristics of two groups: the healthy group (*n* = 100) and the infertile group (*n* = 194). No significant differences in age or body mass index (BMI) were detected between the two groups (Wilcoxon rank-sum test, *P* > 0.05). Specifically, the mean age was 31.7 ± 4.8 years in the infertile group and 32.3 ± 4.52 years in the healthy group (**Figure 1**A). Accordingly, canonical correlation analysis (CCA) revealed that both age and BMI were associated with vaginal microbiota composition (Figure 1B). To further explore the differences within the infertile group, the baseline characteristics of the women who became pregnant (*n* = 95) and those who did not (*n* = 99) were compared. No significant differences were observed in BMI, embryo quality, ART style, or reasons for ART between the two groups. However, the non-pregnant group was significantly older than the pregnant group (*P* < 0.05), with mean ages of 32.7 ± 5.1 years and 30.8 ± 4.3 years, respectively (**Table 1**).

**Table 1:** Baseline characteristics of the infertile group

Characteristic	Group			*P* value
	Overall (*n* = 194)	Non-pregnant (*n* = 99)	Pregnant (*n* = 95)	
**Age (year, mean ± SD)**	31.7 (4.8)	32.7 (5.1)	30.8 (4.3)	0.005
**BMI (kg/m^2^, mean ± SD)**	22.2 (3.3)	22.5 (3.6)	21.8 (3.0)	0.170
**Reason for ART [number (%)]**				0.310
Ovulatory dysfunction	33 (17%)	17 (17%)	16 (17%)	
Tubal disease	71 (37%)	35 (35%)	36 (38%)	
Multiple factor	15 (7.7%)	11 (11%)	4 (4.2%)	
Unknown factor	51 (26%)	26 (26%)	25 (26%)	
Male factor	21 (11%)	10 (10%)	11 (12%)	
Genetic factor	3 (1.5%)	0 (0%)	3 (3.2%)	
**ART style [number (%)]**				0.290
PGT	26 (13%)	14 (14%)	12 (13%)	
ICSI	54 (28%)	32 (32%)	22 (23%)	
IVF	114 (59%)	53 (54%)	61 (64%)	
**Embryo quality [number (%)]**				> 0.990
Good-quality embryo	189 (97%)	96 (97%)	93 (98%)	
Poor-quality embryo	5 (2.6%)	3 (3.0%)	2 (2.1%)	

*Note*: Good-quality embryos include (1) day 3 embryos with a cell count of ≥ 8, evenly sized blastomeres, and a fragmentation rate of < 10% or (2) blastocysts with a grade of ≥ 3BB. BMI, body mass index; ART, assisted reproductive technology; PGT, preimplantation genetic testing; ICSI, intracytoplasmic sperm injection; IVF, *in vitro* fertilization; SD, standard deviation.

As our study focused on the association between the vaginal microbiome and RIF, we then compared the baseline characteristics of the RIF group (*n* = 32, individuals who met the diagnostic criteria for RIF and did not achieve pregnancy in the current transfer cycle), and the non-RIF group (*n* = 83, individuals who did not meet the diagnostic criteria for RIF and successfully achieved pregnancy in the current transfer cycle). No significant differences were observed in embryo quality, ART style, or reasons for ART between the two groups. However, both age and BMI in the RIF group were significantly higher than those in the non-RIF group (*P* < 0.05). Specifically, the mean age and BMI of the non-RIF group were 30.7 ± 4.3 years and 21.7 ± 3.1 kg/m², respectively, while those of the RIF group were 33.5 ± 5.4 years and 23.4 ± 3.6 kg/m², respectively (**Table 2**).

**Table 2:** Baseline characteristics of the RIF and non-RIF groups

Characteristic	Group			*P* value
	Overall (*n* = 115)	Non-RIF (*n* = 83)	RIF (*n* = 32)	
**Age (year, mean ± SD)**	31.5 (4.7)	30.7 (4.3)	33.5 (5.4)	0.011
**BMI (kg/m^2^, mean ± SD)**	22.2 (3.3)	21.7 (3.1)	23.4 (3.6)	0.030
**Reason for ART [number (%)]**				0.340
Ovulatory dysfunction	21 (18%)	15 (18%)	6 (19%)	
Tubal disease	42 (37%)	31 (37%)	11 (34%)	
Multiple factor	6 (5.2%)	2 (2.4%)	4 (12%)	
Unknown factor	31 (27%)	22 (27%)	9 (28%)	
Male factor	12 (10%)	10 (12%)	2 (6.2%)	
Genetic factor	3 (2.6%)	3 (3.6%)	0 (0%)	
**ART style [number (%)]**				0.740
PGT	14 (12%)	10 (12%)	4 (12%)	
ICSI	27 (23%)	18 (22%)	9 (28%)	
IVF	74 (64%)	55 (66%)	19 (59%)	
**Embryo quality [number (%)]**				> 0.990
Good-quality embryo	113 (98%)	81 (98%)	32 (100%)	
Poor-quality embryo	2 (1.7%)	2 (2.4%)	0 (0%)	

*Note*: The RIF group includes individuals who met the diagnostic criteria for RIF and did not achieve pregnancy in the current transfer cycle. The non-RIF group includes individuals who did not meet the diagnostic criteria for RIF and successfully achieves pregnancy in the current transfer cycle. RIF, recurrent implantation failure.

### Alterations in the vaginal microbiota of female patients with infertility

**Figure 2:**
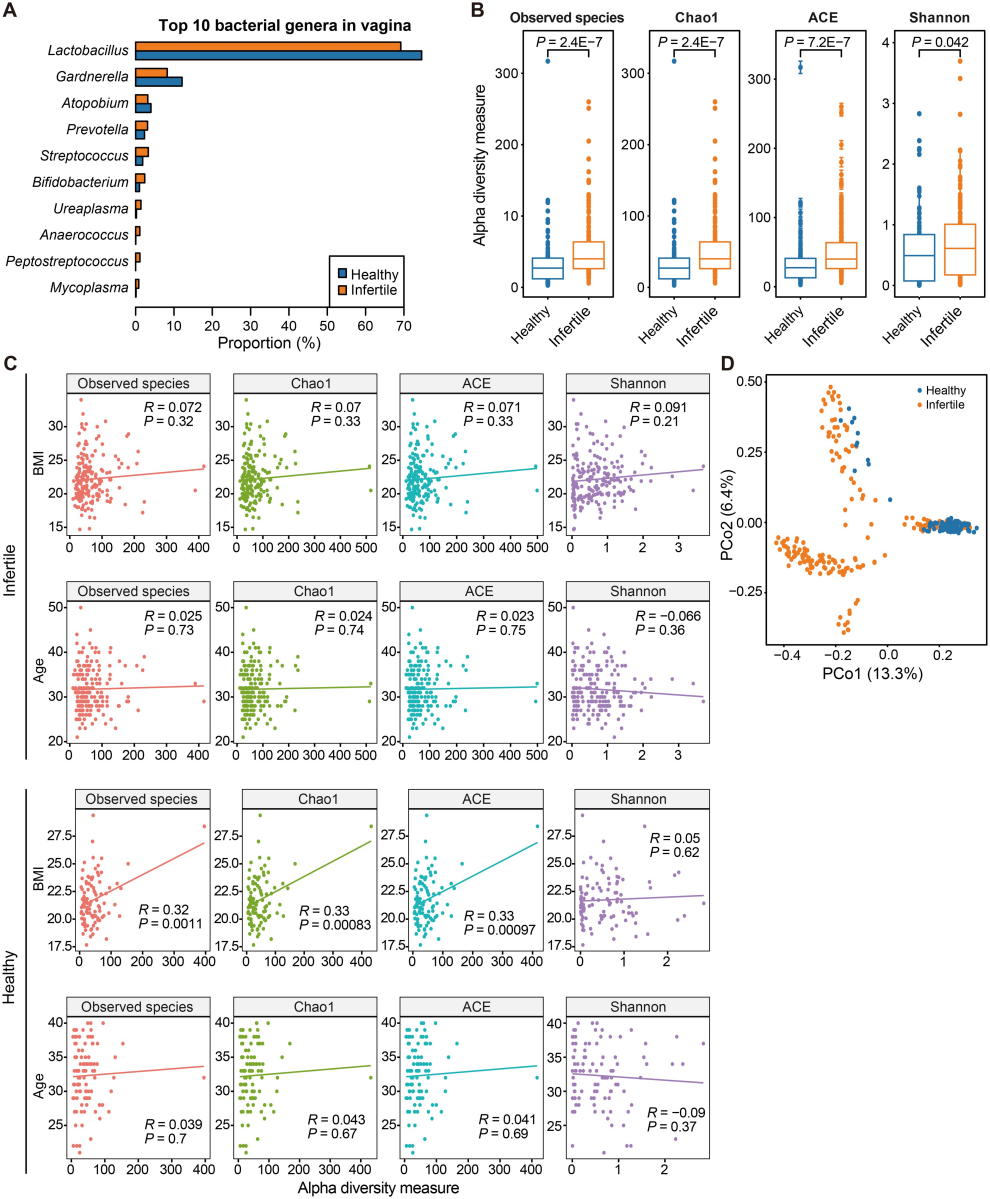
Vaginal microbiota composition and bacterial diversity in infertile patients and healthy controls **A**. Top 10 bacterial genera in the vaginal microbiota of both groups. **B**. Comparison of alpha diversity indices (Observed species, Chao1, ACE, and Shannon) between healthy and infertile groups. Statistical significance was assessed by Wilcoxon rank-sum test, with *P* values indicated above each plot. **C**. Spearman correlation analysis between BMI/age and alpha diversity indices in both infertile and healthy groups. **D**. PCoA plot based on Bray-Curtis distances at the genus level for all taxonomic features of vaginal microbiota between the two groups. PCoA, principal coordinate analysis; PCo, principal coordinate.

16S ribosomal RNA (rRNA) sequencing was performed on vaginal swab samples obtained from 100 healthy controls and 194 infertile patients to explore alterations in the reproductive tract microbiota. The sequencing data revealed ten major bacterial genera (ranked by average abundance) in the vaginal microbiota, including *Lactobacillus*, *Gardnerella*, *Atopobium*, *Prevotella*, *Streptococcus*, *Bifidobacterium*, *Ureaplasma*, *Anaerococcus*, *Peptostreptococcus*, and *Mycoplasma*. Notably, *Lactobacillus*, *Gardnerella*, and *Atopobium* were more abundant in healthy samples, while the remaining genera, such as *Prevotella*, *Streptococcus*, and *Bifidobacterium*, were more abundant in infertile samples. These findings suggest a potential association between the genus abundance in infertile samples and the onset of infertility (**Figure 2**A). Additionally, higher microbial diversity indices (Observed species, Chao1, ACE, and Shannon) were observed in infertile samples compared to healthy controls, indicating that the reproductive tract microbiota in infertile patients is more diverse than that in healthy controls (Figure 2B).

We also examined the correlation between microbial diversity and BMI/age in both healthy and infertile groups. No significant correlation was observed between microbial diversity and age in either group. In the healthy group, all indices except the Shannon index were increased with BMI. In contrast, no significant correlation was found between microbial diversity and BMI in the infertile group. These results suggest that microbiota composition might vary with BMI in healthy individuals but might be influenced by other factors in the infertile group (Figure 2C). Principal coordinate analysis (PCoA) based on microbial abundance further distinguished infertile samples from healthy controls, indicating significant changes in the reproductive tract microbiota in patients with infertility (Figure 2D).

### Infertile women harbor an altered vaginal microbiome compared to healthy controls

**Figure 3:**
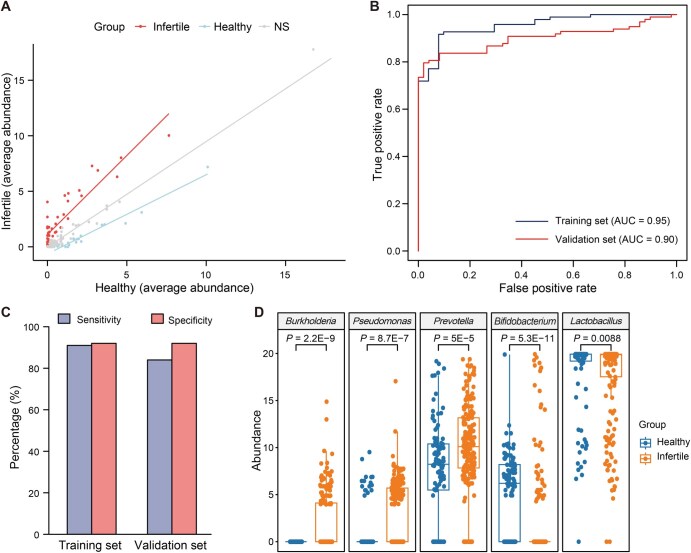
Infertile women harbor an altered vaginal microbiome compared to healthy controls **A**. Average abundance of bacteria at the genus level in infertile and healthy groups. The lines for bacteria enriched in infertile samples (red), healthy samples (light blue), and without statistical significance (light purple) were fitted based on their average abundance. **B**. ROC curves of the naïve Bayesian model for predicting infertility in the training and validation sets. **C**. Sensitivity and specificity of the machine learning model in the training and validation sets. **D**. Genera significantly enriched in infertile (*Burkholderia*, *Pseudomonas*, and *Prevotella*) and healthy (*Bifidobacterium* and *Lactobacillus*) groups, as determined by Wilcoxon rank-sum test (*P* < 0.05). NS, not significant; ROC, receiver operator characteristic; AUC, area under the curve.

To further explore the vaginal microbiota associated with infertility, a differential abundance analysis was conducted by comparing the microbiota composition of infertile patients with that of healthy controls at the genus level. This analysis revealed 45 genera with increased abundance and 43 genera with decreased abundance in the infertile group compared to the healthy controls [*P* < 0.05 and |log_2_ fold change| > 1] (**Figure 3**A). These findings indicate significant alterations in the vaginal microbiota composition of infertile patients.

To assess the predictive capability of the genera with significant differential abundance for infertility, a machine learning model was built. Specifically, 294 samples were randomly divided into a training set (*n* = 147) and a validation set (*n* = 147). Using the abundance data of different bacterial genera in the training set, a naive Bayesian model was constructed. The performance of the model was evaluated based on its ability to predict infertility in the validation set. Encouragingly, the model exhibited an area under the curve (AUC) of over 90% (Figure 3B) as well as sensitivity and specificity exceeding 85% (Figure 3C) in both the training and validation sets. These results suggest that the vaginal microbiota has a strong predictive power for infertility, with high accuracy.

Within the vaginal microbiota of infertile patients, several genera exhibited statistically elevated abundance compared to healthy controls. Specifically, *Burkholderia*, *Pseudomonas*, and *Prevotella* showed significantly higher abundance in the infertile group (*P* < 0.05; Figure 3D). These findings suggest that these bacteria may play a pathogenic role in female infertility. In contrast, the abundance of *Bifidobacterium* and *Lactobacillus* was significantly reduced in the vaginal microbiota of infertile patients (*P* < 0.05; Figure 3D). Given their known protective roles in the vaginal environment [18–20], this reduction may contribute to the development of infertility.

### Vaginal microbiome is associated with RIF, tubal disease, and ART outcomes

**Figure 4:**
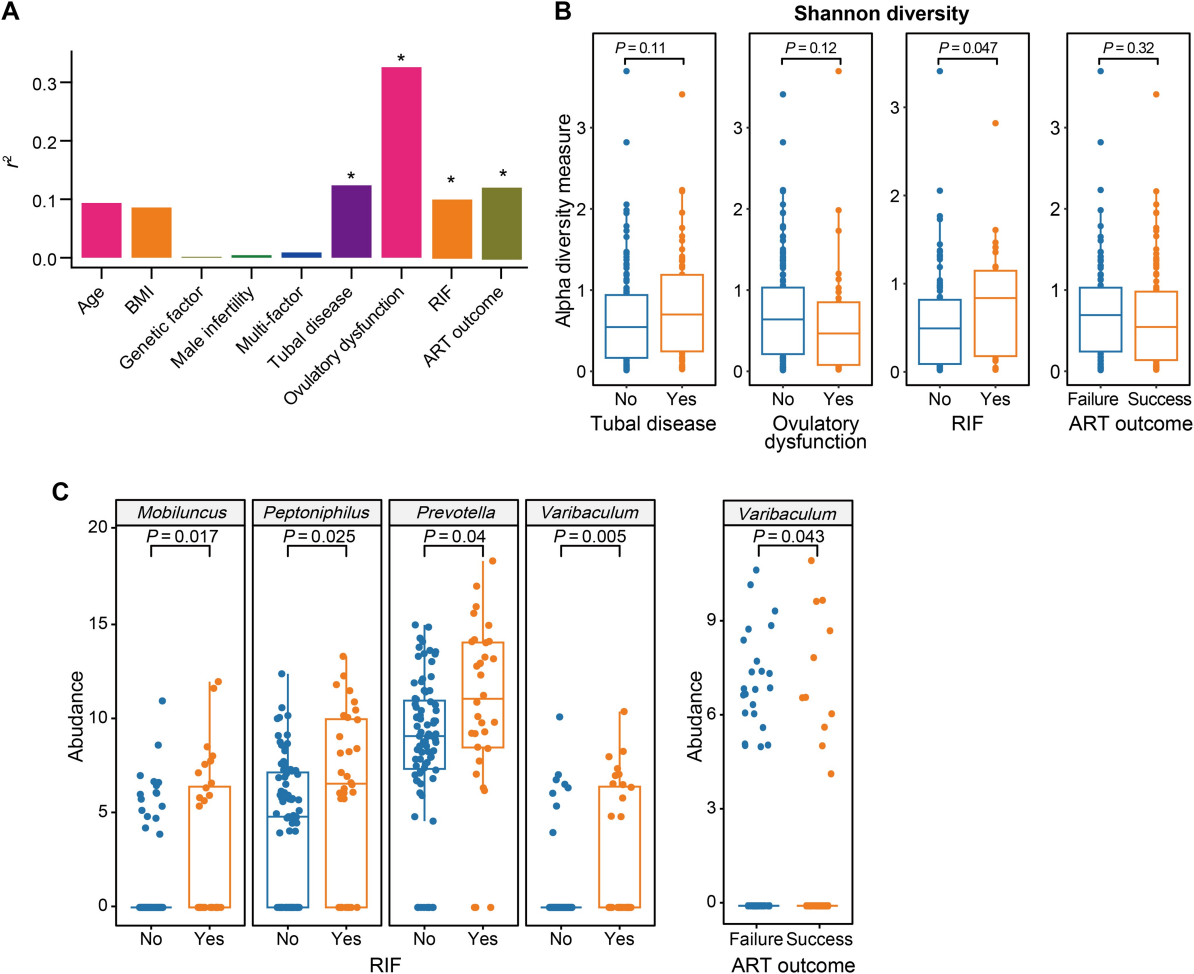
Associations of clinical factors with vaginal microbiome, bacterial diversity, and bacterial genera **A**. Associations of clinical factors with vaginal microbiome, as assessed by CCA. *, *P* < 0.05. **B**. Associations of clinical factors with Shannon diversity. Statistical significance was assessed by Wilcoxon rank-sum test with *P* values indicated above each plot. **C**. Bacterial genera significantly associated with RIF and ART outcomes, as identified by both univariable and multivariable analyses (*P* < 0.05). CCA, canonical correlation analysis; RIF, recurrent implantation failure; ART, assisted reproductive technology.

To further investigate associations between the vaginal microbiome and specific infertility etiologies, ART outcomes, and baseline characteristics (such as age and BMI), we performed CCA. The results revealed strong correlations between the vaginal microbiome and tubal disease, ovulatory dysfunction, RIF, and ART outcomes (*P* < 0.05; **Figure 4**A). Notably, patients with tubal disease, those without ovulatory dysfunction, and those experiencing RIF or ART failure exhibited significantly higher Shannon diversity indices (Figure 4B). Particularly, RIF was found to be significantly associated with Shannon diversity (Wilcoxon rank-sum test, *P* < 0.05). Differential abundance analysis and multivariable linear regression analysis at the genus level further identified specific bacterial genera that were altered in the RIF and ART failure groups compared to their respective control groups. By intersecting these differential genera with those observed between infertile patients and healthy controls, we identified four genera (*Mobiluncus*, *Peptoniphilus*, *Prevotella*, and *Varibaculum*) that were significantly enriched in the RIF group. Additionally, *Varibaculum* was abundant in the ART failure group (Figure 4C). These findings suggest that the vaginal microbiome is more closely associated with RIF and ART outcomes, providing further evidence for the role of vaginal microbiota in infertility etiologies and treatment outcomes.

### Pathway analysis interprets associations of vaginal microbiome with RIF, tubal disease, and ART outcomes

**Figure 5:**
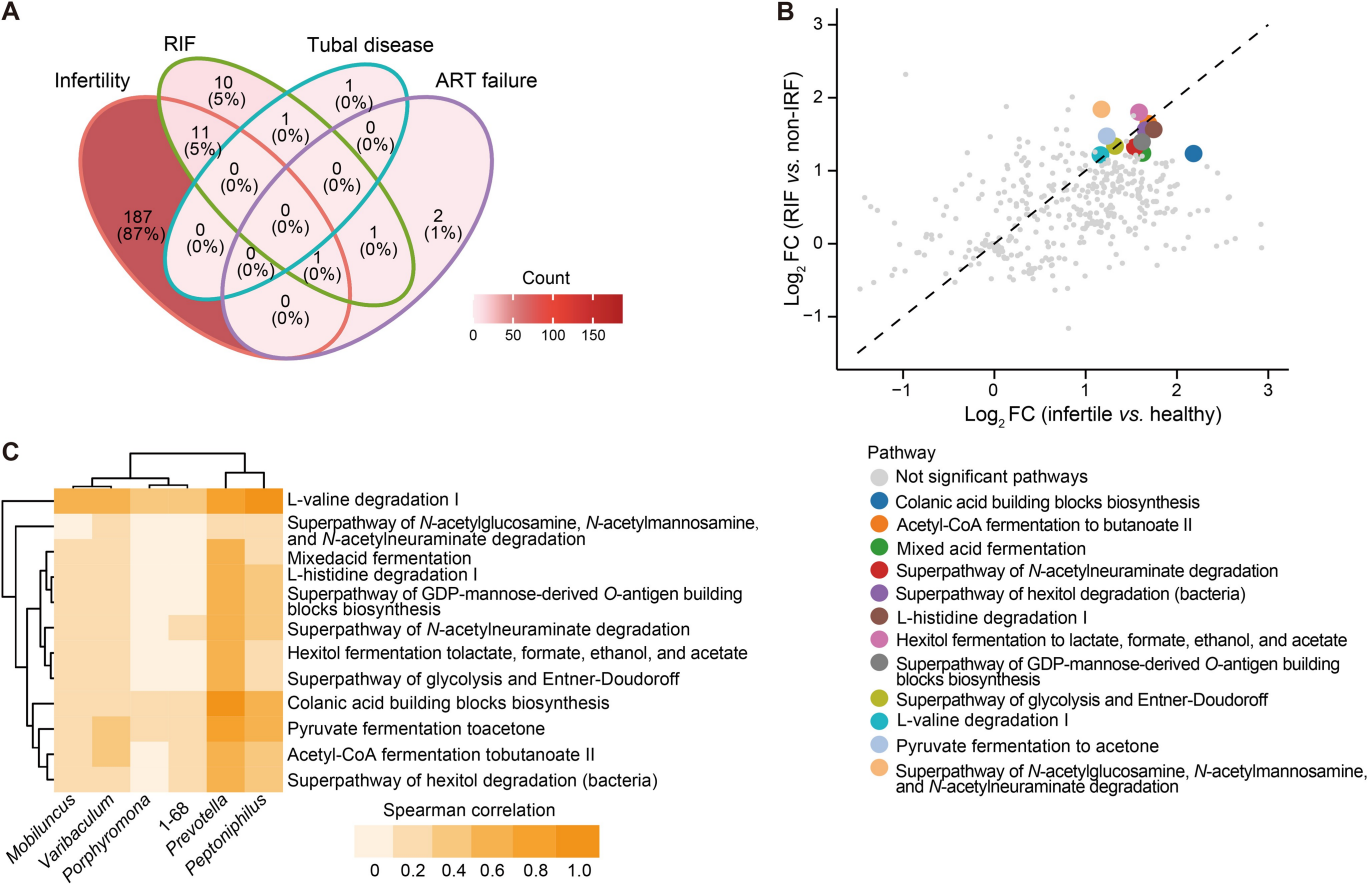
Associations of clinical factors with pathways inferred by vaginal microbiome **A**. Venn diagram displaying the intersections of pathways associated with clinical factors. Color corresponds to the pathway count. **B**. Eleven pathways significantly associated with both infertility and RIF (*P* < 0.05). **C**. Spearman correlation of the activities of 11 pathways and the abundance of bacterial genera associated with infertility, RIF, and ART outcomes. FC, fold change.

To further explore the potential molecular mechanisms underlying female infertility and ART outcomes, we employed the Phylogenetic Investigation of Communities by Reconstruction of Unobserved States (PICRUSt) algorithm to predict metabolic pathway activities based on the microbiomic data obtained from different samples. By comparing the pathway activities among different groups, we identified pathways associated with infertility, RIF, tubal disease, and ART failure (**Figure 5**A). Surprisingly, we identified 11 pathways that were associated with both RIF and infertility. These pathways included colanic acid building blocks biosynthesis, acetyl-CoA fermentation to butanoate II, mixed acid fermentation, superpathway of *N*-acetylneuraminate degradation, superpathway of hexitol degradation (bacteria), L-histidine degradation I, hexitol fermentation to lactate, formate, ethanol, and acetate, superpathway of GDP-mannose-derived *O*-antigen building blocks biosynthesis, superpathway of glycolysis and Entner-Doudoroff, L-valine degradation I, and pyruvate fermentation to acetone. Notably, one additional pathway, involving the degradation of *N*-acetylglucosamine, *N*-acetylmannosamine, and *N*-acetylneuraminate, was found to be simultaneously associated with RIF, ART failure, and infertility (*P* < 0.05; Figure 5B).

Furthermore, these pathways were closely linked to the regulation of vaginal pH, a critical factor in maintaining the balance of the vaginal microbiome. Correlation analysis revealed a positive association between the genera associated with infertility, RIF, and ART outcomes and the activities of these pathways (Figure 5C). Notably, *Prevotella*, one of the genera identified in our previous analysis, showed stronger correlations with multiple pathways compared to other genera. This suggests that *Prevotella* may play a crucial role in processes related to infertility, RIF, and ART outcomes through these metabolic pathways.

## Discussion

There is growing evidence that microorganisms are closely associated with the health of female reproductive tract. The vaginal microbiota of women of childbearing age is dominated by *Lactobacillus* and maintains a dynamic balance. When affected by a variety of endogenous and exogenous factors, the relative abundance of *Lactobacillus* and other vaginal microbial components will change, resulting in vaginal dysbiosis. Such dysbiosis is the main cause of bacterial vaginosis (BV). Previous studies have demonstrated a correlation between BV and infertility [21,22]. A systematic review and meta-analysis revealed that abnormal microbiota was observed in 39% of infertile patients, that the estimated prevalence of BV in infertile women was 19%, and that the incidence of BV was significantly higher in infertile women than in pregnant women in the same population [23]. These findings suggest that dysbiosis of the vaginal microbiota is closely associated with the occurrence of infertility. Using 16S rRNA gene sequencing, our previous study revealed for the first time the distribution characteristics of the reproductive tract microbiota in healthy Chinese women, as well as their functional roles in female infertility in the Chinese population [24]. In this study, we aimed to further expand our knowledge of vaginal microecosystems in the context of female infertility and ART outcomes. By comparing the vaginal microbiomes of infertile women with those of healthy women, we elucidated the microbial composition of the infertile population and its association with ART outcomes in the Chinese population.

First, in our study, we found that both age and BMI are related to vaginal microbiota composition, with a stronger correlation observed in healthy groups than in infertile groups. This suggests that the vaginal microbiota of infertile patients is also affected by other factors. As we know, the causes of infertility are complex and varied, and approximately 30% of female infertility cases remain unexplained and are labeled “unexplained infertility (UI)” [25]. There is a growing interest in exploring the reproductive tract microbiota as a potential novel strategy to enhance ART outcomes among patients with UI. The diagnosis of UI inherently possesses a degree of ambiguity, which presents challenges in treatment and management. Recent studies have indicated that UI is associated with factors such as endometrial receptivity [26], endocrine factors [27], and immune factors [28]. Vaginal dysbiosis has the potential to activate the host immune system, triggering an immune response and the production of multiple inflammatory cytokines. This disruption can alter the levels of various immune factors and immune cells within the body, thereby inducing a state of chronic inflammation, which may ultimately contribute to the development of female infertility [29,30]. However, the current understanding of the impact of reproductive tract microbiota on UI is still limited by a scarcity of information.

Second, we developed a machine learning model to evaluate the predictive capability of genera that exhibited significant abundance differences related to infertility, offering new insights into the potential role of the vaginal microbiota in female infertility. Our analysis revealed higher microbial diversity in the infertile group, mainly characterized by an increased abundance of *Burkholderia*, *Pseudomonas*, and *Prevotella*, alongside a reduction in *Bifidobacterium* and *Lactobacillus*. As we all know, *Lactobacillus* plays a crucial role in maintaining the health of the female reproductive tract, by inhibiting the adhesion of other bacteria to epithelial cells, and producing lactic acid, which kills or suppresses the growth of numerous other bacteria [31,32]. The predominance of *Lactobacillus* in the vagina facilitates a favourable environment for conception and embryo implantation [18]. A study by Moreno and colleagues [33], demonstrated that women with a uterine endometrial microbiome dominated by non-*Lactobacillus* species had a significantly lower embryo implantation rate (23.1%) than those with a microbiome dominated by *Lactobacillus* species (60.7%, *P =* 0.02). Previous studies have demonstrated a correlation between low *Lactobacillus* abundance and infertility, which align with the results of our study [32,34].

In addition, RIF, which affects 15%–20% of individuals undergoing *in vitro* fertilization and embryo transfer (IVF-ET) cycles [14], is indeed a complex issue that requires careful consideration. In this study, both univariable and multivariable analyses revealed a close association between RIF and the reproductive tract microbiota. Additionally, the multivariable linear regression model analysis identified four genera (*Mobiluncus*, *Peptoniphilus*, *Prevotella*, and *Varibaculum*) significantly distributed in the RIF group. Among these microbes, *Prevotella* is associated with various pathways and has attracted much attention from scholars. *Prevotella* is a Gram-negative, anaerobic, and immobile bacillus, and recent studies have shown that its high concentration is associated with infertility [35]. Additionally, in many patients with intrauterine adhesions (IUA), a significant reduction in *Lactobacillus* in the vagina has been observed, along with excessive growth of *Gardnerella* and *Prevotella*. IUA can lead to abnormal menstruation, infertility, or recurrent miscarriage [36–38]. Furthermore, the relative abundance of *Prevotella* is higher in pregnant women with PPROM than in those who deliver at term [39]. *Mobiluncus* is a specialized anaerobic, Gram-variable/Gram-negative, curved bacterium in the vaginal microbiota that is strongly associated with BV [40–42]. Kitaya et al. [43] reported that the levels of *Gardnerella* and *Burkholderia* were elevated in the reproductive tracts of women with RIF compared to those without RIF.

Furthermore, we observed significantly higher abundance of *Varibaculum* in the ART failure group compared to the successful group. *Varibaculum* has been reported to be associated with both bladder cancer and prostate cancer [44,45], providing further evidence of its potential role in various pathological conditions. In addition, evidence from a previous report suggests an association between *Varibaculum* syndrome and male infertility [46]. This association is particularly interesting given that male infertility is a significant factor contributing to overall infertility issues. In our study, we identified *Varibaculum* overgrowth as an independent risk factor affecting ART outcomes. This finding aligns with previous reports of the association between *Varibaculum* and infertility and suggests that it may play a role in the success or failure of ART procedures. However, the precise mechanisms by which *Varibaculum* affects infertility and ART outcomes remain unclear.

Finally, to gain deeper insights into the potential molecular mechanisms underlying female infertility and ART outcomes, we conducted molecular pathway prediction in this study. Our study suggests that *Prevotella* may play a crucial role in processes such as infertility, RIF, and ART outcomes through multiple metabolic pathways. Previous studies have shown that the enrichment of *Prevotella* is related to the degradation of *N*-acetylneuraminate (*N*-acetylneuraminic acid) [47], leading us to speculate that *Prevotella* may contribute to infertility and RIF by regulating the degradation of *N*-acetylneuraminate. *N*-acetylneuraminate is an important sialic acid that exists on the surface of cells and glycoproteins. It is involved in a variety of biological processes, including cellular signal transduction and immune regulation. The presence of *N*-acetylneuraminate on sperm and endometrial cells is essential for successful fertilization and implantation. However, when *N*-acetylneuraminate is degraded or modified, it may lead to the production of anti-*N*-acetylneuraminate antibodies, potentially inducing inflammation and immune response which adversely affect reproductive health and thus impair fertility [48]. In addition, it has been reported that the acetyl-CoA fermentation to butanoate II pathway is closely related to male infertility, although there is a lack of related microorganisms [49]. In short, the current understanding of the role of *Prevotella* in infertility and RIF remains limited and is not yet well-supported by more literature. Perhaps more animal experiments can be conducted to verify the mechanism of action between *Prevotella* and the abovementioned metabolic pathways in the future.

However, the present study still has some limitations. First, the underlying mechanisms remain unexplored and require further investigation. Second, as a single-center study, the results may not be universally applicable. Third, the small sample size available may have impacted the ability to detect significant differences in vaginal microbial diversity among patients with various causes of infertility in terms of pregnancy outcomes following ART. It is possible that with a larger sample size, more subtle differences in microbial diversity may be identified, which could provide additional insights into the relationship between the vaginal microbiota and ART success. Future research with a larger sample size could verify the machine learning model and the clinical relevance of the bacteria taxa identified in this study.

Given the potential influence of the FGT microbiome on embryo implantation and pregnancy outcomes, it is crucial to consider its role in ART success. Modifying the FGT microbiome may be a promising approach for improving ART outcomes in specific cases. Therefore, it is advisable for infertile women to undergo reproductive tract microorganism screening prior to ART and receive exogenous *Lactobacillus* supplementation in cases of deficiency [50,51]. In addition, for some infertile patients with overgrowth of pathogenic bacteria (*e.g.*, *Prevotella* and *Pseudomonas*), targeted antibiotic treatment before *Lactobacillus* supplementation may be beneficial. This strategy may help increase the chances of successful embryo implantation and pregnancy, ultimately leading to more successful ART outcomes.

## Conclusion

The current study offers valuable insights into the variations in the vaginal microbiota composition between healthy and infertile women, shedding light on the potential influence of the vaginal microbiota on infertility and ART outcomes. This study identified specific genera associated with RIF within the infertile population. Future research should continue to expand upon these findings, aiming to uncover additional microbial biomarkers and develop innovative therapeutic interventions to effectively address the challenges posed by infertility.

## Materials and methods

### Sample collection

In this study, a total of 194 women diagnosed with infertility at the Reproductive Medicine Center of Shanghai Changzheng Hospital, China between November 2018 and November 2021 were enrolled as the infertile group. Additionally, 100 healthy women who underwent routine physical examinations at the Physical Examination Center of the same hospital were recruited as the healthy control group.

The inclusion criterion for the infertile group was regular unprotected sexual activity for at least one year without achieving pregnancy. The inclusion criterion for the healthy group was a history of childbirth with no record of infertility or other gynecological diseases.

The exclusion criteria for both groups included the presence of an intrauterine device (IUD), vaginal inflammation, any acute inflammatory condition, suspected cervical or endometrial neoplasia, and endocrine or autoimmune disorders. Subjects were also excluded if they had recently used hormones, antibiotics, or vaginal medications; undergone cervical treatment, endometrial biopsy, IUD removal, or hysteroscopy within the past week; engaged in douching within five days; or had sexual activity within 48 h prior to sampling. Additionally, none of the participants were pregnant, lactating, or menstruating at the time of sampling.

Data on age, current residence, menstrual history, and fertility history were collected from all participants. Nulliparous subjects were asked about their pregnancy plans. Vaginal secretions were collected from the posterior fornix region of participants in both groups. These samples were then immediately frozen in liquid nitrogen, stored at −80°C, and transported on dry ice to BGI-Shenzhen for further analysis.

### DNA extraction, 16S rRNA amplicon sequencing, and data processing

Genomic DNA was extracted from vaginal secretion samples and subjected to polymerase chain reaction (PCR) amplification of high-quality DNA. More accurate distance-based clustering of reads was achieved by using V3–V4 primers and PCR premixes with targeted primers for these regions. The library was prepared with 2× Phanta Max Master Mix (Catalog No. P525, Vazyme Biotech, Nanjing, China), and the V3–V4 hypervariable region of the bacterial 16S rDNA was amplified with the degenerate PCR primers 338F (5′-AC TCCTACGGGAGGCAGCAG-3′) and 806R (5′-GACTA CHVGGGTWTCTAAT3′). PCR amplification was performed in a 50-µl reaction mixture containing 30 ng of template DNA and fusion PCR primers. The PCR cycling conditions were as follows: 95°C for 3 min; 30 cycles of 95°C for 15 s, 56°C for 15 s, and 72°C for 45 s; and a final extension at 72°C for 5 min. The PCR products were purified with DNA magnetic beads (Catalog No. LB00V60, BGI Genomics, Shenzhen, China). The validated libraries were sequenced on an Illumina HiSeq platform (BGI, Shenzhen, China) following the standard Illumina pipelines, generating 2 × 250 bp paired-end reads. The raw sequencing data were processed as follows [52]: (1) reads with an average Phred quality score below 20 within a 30-bp sliding window were truncated, and the resulting reads with length shorter than 75% of their original length after truncation were removed; (2) reads contaminated by adapter sequences were removed; (3) reads containing ambiguous bases (N bases) were removed; and (4) low-complexity reads were removed.

### Amplicon sequence variant and taxonomy analyses

The cleaned 16S rRNA sequencing data were processed using the DADA2 package in R [53], which offers a precise and sensitive pipeline for amplicon sequencing analysis. The DADA2 pipeline proceeded as follows: (1) clean reads were filtered and trimmed, which involved discarding reads at the first instance of a quality score less than or equal to 2; (2) duplicated sequences in the Fast-Accompanied Sequence Text with Quality (FASTQ) files were removed; (3) paired reads were merged; (4) error rates were evaluated, and sample composition was inferred with these error rates; (5) a sequence table was constructed; (6) chimeras were removed; and (7) taxonomy was assigned using the naive Bayesian classifier method with the Ribosomal Database Project (RDP) 16S rRNA reference database.

### Normalization of bacterial relative abundance

For each sample, the raw count of each bacterial taxon was divided by the total read count to obtain its relative abundance at the genus or species level. This proportion was used as the normalized abundance. In addition, taxa that accounted for less than 0.5% of the total reads in a sample were merged into “other”. Only taxa that accounted for more than 0.5% of the total reads in at least two samples were retained for further data analysis.

### Differential abundance analysis

The bacteria with differential abundance between two given groups were identified by both univariable and multivariable analyses implemented in the R microbiomeMarker package [54]. In addition, the influence of confounding factors on bacterial abundance was assessed using analysis of variance (ANOVA).

### Diversity analysis

The alpha diversity was analyzed with the mothur package [55]. Based on the operational taxonomic unit (OTU) table, we calculated the Observed species, Shannon index, Chao1 index, and ACE value to estimate the within-sample diversity. PCoA was also performed on the sequencing data. The Chao1 index and Observed species reflect the abundance of OTUs in the samples, whereas the Shannon index reflects the diversity of OTUs in the samples. The beta diversity analysis [56–58] was employed to evaluate differences in microbial composition between samples.

### Machine learning model for infertility prediction

To accurately predict infertility status based on bacterial abundance, we first randomly divided 294 samples into a training set (*n* = 147) and a validation set (*n* = 147). Subsequently, we selected 57 taxa that exhibited differential abundance between infertile and healthy groups in the training set (*P* < 0.05), and trained the model using the abundance profile of these taxa using naïve Bayesian classifier. The receiver operator characteristic (ROC) curve and the corresponding AUC value were calculated by the R ROCR package [59]. The model construction and evaluation procedures were implemented by the R e1071 package (https://cran.r-project.org/web/packages/e1071/index.html).

### Functional prediction of microbiota

PICRUSt2 was used to predict the functional potential of the microbiota based on marker gene sequencing profiles [60]. Functional prediction provides critical biological insights into microbial diversity, and can link species to their metabolic capabilities to obtain the overall functional distribution within a community.

### Differential abundance analysis

Differential abundance of microbiota between two groups was assessed using the Wilcoxon rank-sum test. *P* values were adjusted using the Benjamini–Hochberg method. For multiple comparisons, the Kruskal‒Wallis test was employed.

## Ethical statement

The study was approved by the Ethics Committee of Shanghai Changzheng Hospital, China (Approval No. 2018SLYS2). All participants were informed of the study’s purpose and provided written informed consent.

## Data Availability

The metagenomic sequencing data generated in this study have been deposited in the Genome Sequence Archive [61] at the National Genomics Data Center (NGDC), China National Center for Bioinformation (CNCB) (GSA: PRJCA032884), and are publicly available at https://ngdc.cncb.ac.cn/gsa.

## CRediT author statement

**Xiuju Chen:** Methodology, Data curation, Formal analysis, Visualization, Writing – original draft. **Yanyu Sui:** Resources, Investigation, Data curation, Validation. **Jiayi Gu:** Resources, Data curation, Validation. **Liang Wang:** Conceptualization, Writing – review & editing, Supervision. **Ningxia Sun:** Conceptualization, Writing – review & editing, Supervision, Funding acquisition. All authors have read and approved the final manuscript.
